# Debra, a Protein Mediating Lysosomal Degradation, Is Required for Long-Term Memory in *Drosophila*

**DOI:** 10.1371/journal.pone.0025902

**Published:** 2011-10-03

**Authors:** Benjamin Kottler, Aurélie Lampin-Saint-Amaux, Daniel Comas, Thomas Preat, Valérie Goguel

**Affiliations:** Genes and Dynamics of Memory Systems, Neurobiology Unit, CNRS, ESPCI, Paris, France; University of Missouri, United States of America

## Abstract

A central goal of neuroscience is to understand how neural circuits encode memory and guide behavior changes. Many of the molecular mechanisms underlying memory are conserved from flies to mammals, and *Drosophila* has been used extensively to study memory processes. To identify new genes involved in long-term memory, we screened *Drosophila* enhancer-trap P(Gal4) lines showing Gal4 expression in the mushroom bodies, a specialized brain structure involved in olfactory memory. This screening led to the isolation of a memory mutant that carries a P-element insertion in the *debra* locus. *debra* encodes a protein involved in the Hedgehog signaling pathway as a mediator of protein degradation by the lysosome. To study *debra*'s role in memory, we achieved *debra* overexpression, as well as *debra* silencing mediated by RNA interference. Experiments conducted with a conditional driver that allowed us to specifically restrict transgene expression in the adult mushroom bodies led to a long-term memory defect. Several conclusions can be drawn from these results: i) *debra* levels must be precisely regulated to support normal long-term memory, ii) the role of *debra* in this process is physiological rather than developmental, and iii) *debra* is specifically required for long-term memory, as it is dispensable for earlier memory phases. *Drosophila* long-term memory is the only long-lasting memory phase whose formation requires *de novo* protein synthesis, a process underlying synaptic plasticity. It has been shown in several organisms that regulation of proteins at synapses occurs not only at translation level of but also via protein degradation, acting in remodeling synapses. Our work gives further support to a role of protein degradation in long-term memory, and suggests that the lysosome plays a role in this process.

## Introduction

*Drosophila melanogaster* constitutes a useful model to study the molecular basis underlying memory processes. Its brain, despite its small size, is highly organized and exhibits specialized structures. Furthermore, many of the mechanisms inherent in memory are conserved from flies to mammals [1]. Studies in *Drosophila* combine the use of powerful genetic tools together with the possibility of analyzing a large repertoire of behaviors. The genetic basis of olfactory learning and memory has been studied for more than 30 years in *Drosophila*, providing insights into some of the genes involved in short-term and long-term memory formation.

Aversive olfactory memory studies generally rely on classical conditioning of an odor-avoidance response. In this paradigm, groups of flies are successively exposed to two distinct odors, only one of which is accompanied by electric shocks [2], [3]. Memory scores are determined by placing the flies in the center of a T-maze where they are simultaneously exposed to the two odors during one minute [2]. Depending on the training protocol, different types of memory can be measured [4]. Short-term memory (STM) and anaesthesia-resistant memory (ARM) are formed after one cycle of training. STM is a labile memory phase sensitive to cold shock anaesthesia that lasts for a few hours. In contrast, ARM is a consolidated form of memory resistant to cold shock that can last for days [5]. Long-term memory (LTM) is also a form of consolidated memory, but unlike ARM, its formation is sensitive to an inhibitor of cytoplasmic protein synthesis, indicating that *de novo* protein synthesis is required [4]. LTM is generated after spaced-conditioning consisting of repeated training sessions, each separated by a rest period. LTM is generally thought to occur through changes in synaptic efficacy produced by a restructuring of synapses [6].

The requirement for *de novo* gene expression during LTM formation has been widely observed in a number of different model systems [7]. The cAMP response element-binding protein is an LTM-specific regulator of gene expression in *Drosophila* [8], [9] and in other species [10], [11]. Several other transcription regulators are required for proper LTM including Adf-1 [12] and Stat92E [13] in *Drosophila*, and CCAAT/enhancer-binding protein, Zif-268, AP-1, and NF-kB in mammals [10]. The Notch signaling receptor has also been implicated in LTM [14], [15]. In addition to transcription, local control of translation [16], and proteases are as well involved in *Drosophila* LTM [17], [18]. Crammer, a protein required for LTM [17], has been shown to inhibit Cathepsin L, a protease that could be involved in lysosome function [19].

A large collection of evidence indicates that mushroom bodies (MBs) play a pivotal role in olfactory memory [20], [21], [22]. The MBs form a bilaterally symmetrical structure in the central brain and consist of approximately 4,000 neurons called Kenyon cells. Three types of Kenyon cells (α/β, α'/β', and γ) project their axons ventrally to form the peduncle that splits into five lobes, two vertical (α and α') and three median (β, β', and γ) [23]. The lobes are assumed to be the synaptic output region of the MBs [24]. In addition, neurons of the lobes are targeted by multiple inputs [22].

Many genes required for LTM have been shown to be expressed in the MBs [1], prompting us to analyze enhancer-trap P(Gal4) lines showing Gal4 expression in the MBs to characterize new LTM mutants. In this report we identify *debra*, a gene involved in protein degradation by the lysosome, as being specifically required for LTM.

## Results

### Identification of a new LTM mutant

We screened 91 enhancer-trap P(Gal4) lines showing adult MB expression for performance defects after spaced conditioning toward aversive olfactory memory. These lines were selected from the collection of D. Armstrong (www.fly-trap.org) and from our own collection [25]. At least ten groups of flies were trained and tested for each line carrying a homozygous P-element insertion. Eleven strains showed a score significantly lower than the wild-type. After outcrossing to a *w^1118^* line with a CS background, 8 lines retained a LTM defect, but 6 of them also displayed either an apparent STM or ARM defect (data not shown). We report here the characterization of a new LTM mutant, the strain *72Y*.

**Figure 1:**
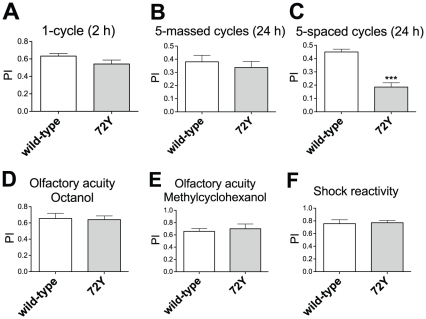
Behavioral analysis of the *72Y* enhancer-trap mutant. (A) STM analysis. Performance indices (PI) were measured 2 hrs after a single conditioning cycle (*t* test, *p* = 0.0913, *n*≥10). (B) ARM analysis. PI were measured 24 hrs after five massed conditioning cycles (*t* test, *p* = 0.5298, *n*≥12). (C) LTM analysis. PI were measured 24 hrs after five spaced conditioning cycles (*t* test, *p*<0.0001, *n*≥17). Olfactory acuity for (D) octanol (*t* test, *p* = 0.8645, *n* = 16) and (E) methylcyclohexanol (*t* test, *p* = 0.6309, *n*≥12). (F) Shock sensitivity (*t* test, *p* = 0.8550, *n* = 10). Bars indicate mean ± SEM.

On testing 2 hrs after a single conditioning cycle, we did not observe significant differences in memory scores for the enhancer-trap *72Y* flies compared to wild-type CS flies, showing that neither STM nor 2 hrs ARM are affected (Figure 1A). To further analyze ARM, consolidated memory was assessed using a reinforced training protocol. After massed conditioning, comprising five consecutive repeated cycles of training, scores at 24 hrs were not significantly different between *72Y* and wild-type flies (Figure 1B), showing that ARM is not affected. In contrast, memory analysis after five spaced conditioning cycles revealed a strong LTM defect for *72Y* flies (Figure 1C). We next verified that *72Y* flies perceived normally the stimuli used for conditioning. Their response to each odor after electric shock exposure was not impaired (Figure 1D and E), and neither was their ability to escape electric shocks (Figure 1F). We conclude that *72Y* flies are specifically impaired for LTM.

**Figure 2:**
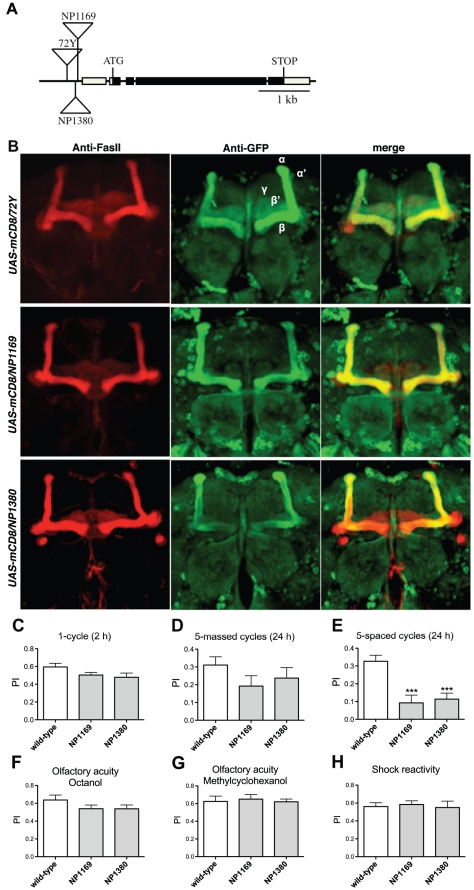
The *dbr* locus is specifically required for LTM. (A) Molecular map of the *dbr* locus. The *72Y* P-element is inserted 353 bp upstream of the *dbr* transcriptional start site, those from the *NP1169* and *NP1380* lines are inserted 90 bp and 149 bp upstream of the *dbr* transcriptional start site, respectively. Boxes, genomic DNA corresponding to exons; black boxes, coding sequences. (B) *72Y, NP1169* and *NP1380* enhancer-trap expression pattern. Freshly dissected brains from *UAS-mCD8-GFP/72Y, UAS-mCD8-GFP/NP1169* and *UAS-mCD8-GFP/NP1380* flies were incubated with anti-FasII antibodies to label the α/β and γ neurons (red), and anti-GFP antibodies (green). (C-H) *NP1169* and *NP1380* behavioral analysis. (C) STM analyzed 2 hrs after a single conditioning cycle is not affected (*t* test, *p*>0.05, *n* = 10). (D) ARM analyzed 24 hrs after five massed conditioning cycles is not affected (*t* test, *p*>0.05, *n*≥15). (E) LTM analyzed 24 hrs after five spaced conditioning cycles is impaired (*t* test, *p*<0.001, *n*≥11). Olfactory acuity for (F) octanol (*t* test, *p*>0.05, *n*≥14) and (G) methylcyclohexanol (*t* test, *p*>0.05, *n* = 8). (H) Shock sensitivity (*t* test, *p*>0.05, *n* = 9). Bars indicate mean ± SEM.

We identified the insertion site of the P-element in *72Y* flies by PCR-rescue and found that the closest gene is *debra* (*dbr*, CG11371). *dbr* encodes a 1007 aa protein that has been described as a mediator of protein polyubiquitination and degradation [26]. The P-element inserted in *72Y* is localized 353 bp upstream of the *dbr* transcriptional start site (Figure 2A). No other gene has been described within 6 kb of the P-insertion (Flybase). We selected two additional lines carrying a P-element inserted into the *dbr* locus. The *NP1169* and *NP1380* P insertions are located 90 bp and 149 bp upstream of the *dbr* transcriptional start site, respectively (Figure 2A). Immunohistochemistry experiments were conducted to analyze GFP expression in *UAS-mCD8-GFP/72Y, UAS-mCD8-GFP/NP1169* and *UAS-mCD8-GFP/NP1380* brains. Expression pattern revealed in the different genotypes a similar strong GFP staining in the α/β neurons, and a weak one in the α'/β' and γ neurons (Figure 2B).

Homozygous *NP1169* and *NP1380* mutants were trained with the different protocols and their memory tested. Both lines displayed STM and ARM that did not significantly differ from wild-type (Figure 2C and D), while LTM was affected (Figure 2E). We verified that *NP1169* and *NP1380* mutant flies perceived normally the stimuli used for conditioning (Figure 2F, G and H). These results further show that disruption of *dbr* expression leads to an LTM-specific defect.

### Behavioral analysis of *dbr* silencing in the adult MBs

**Figure 3:**
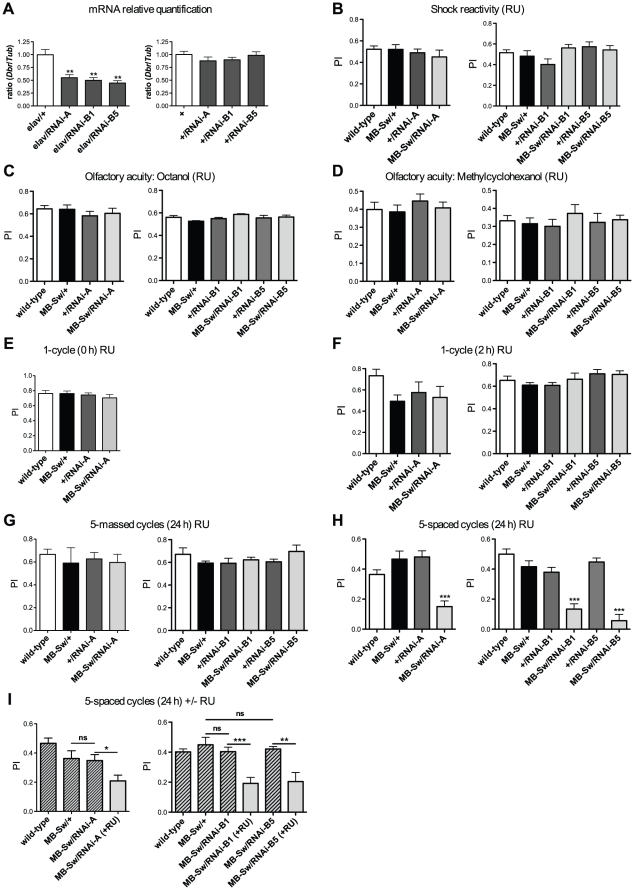
*dbr* RNAi expression in the adult MBs impairs LTM formation. (A) *dbr* mRNA expression analyses. Total RNA was extracted from fly heads, submitted to DNase treatment, and further reverse transcribed with oligo(dT) primers. Resulting cDNA was quantified by PCR using tubulin (*Tub*) expression as a reference. Results are shown as ratios relative to the values observed for *elav/+* (left panel, *t* test, *p*<0.01, n≥5) and wild-type (+, right panel, *t* test, *p*>0.05, n≥4) control flies. (B–I) Behavioral analyses. To silence *dbr* expression in the adult MBs, flies were fed with RU for 48 hrs prior to conditioning and in (G–I), also for 24 hrs before testing. (B) Shock reactivity (ANOVA, left panel, *p* = 0.604, *n* = 9; right panel, *p* = 0.1049, *n*≥8). (C) Olfactory acuity for octanol (ANOVA, left panel, *p* = 0.8099, *n* = 10; right panel, *p* = 0.0891, *n*≥8). (D) Olfactory acuity for methylcyclohexanol (ANOVA, left panel *p* = 0.9488, *n*≥8; right panel, *p* = 0.1633, *n*≥8). (E) Learning analysis after a single conditioning cycle (ANOVA, *p* = 0.5396, *n* = 8). (F) STM analysis 2 hrs after a single conditioning cycle (ANOVA, left panel, *p* = 0.6264, *n*≥10; right panel, *p* = 0.0472, *n*≥8, the Newman-Keuls post-test is only significant for the *MB-Sw/RNAi-B1* vs *MB-Sw/+* pair, *q* = 4.474). (G) ARM analysis 24 hrs after five massed conditioning cycles (ANOVA, left panel, *p* = 0.5088, *n* = 16; right panel, *p* = 0.7635, *n*≥13). (H) LTM analysis 24 hrs after five spaced conditioning cycles (ANOVA, left panel, *p*<0.0001, *n*≥21; right panel, *p*<0.0001, *n*≥14). (I) In the absence of RU, LTM is not affected. Flies were either raised on regular medium (hatched bars) or fed with RU for 48 hrs prior to training, and until testing (+RU) (gray bars). Scores were measured 24 hrs after a five-spaced cycle conditioning (*t* test: left panel, *MB-Sw/RNAi-A* vs *MB-Sw/+*, *p* = 0.8335, *MB-Sw/RNAi-A* vs *MB-Sw/RNAi-A* (+RU), *p* = 0.0222, *n* = 12; right panel, *MB-Sw/RNAi-B1* vs *MB-Sw/+*, *p* = 0.4432, *MB-Sw/RNAi-B1* vs *MB-Sw/RNAi-B1* (+RU), *p* = 0.0005, *n*≥8 ; *MB-Sw/RNAi-B5* vs *MB-Sw/+*, *p* = 0.5989, *MB-Sw/RNAi-B5* vs *MB-Sw/RNAi-B5* (+RU), *p* = 0.0015, *n*≥8). Bars indicate Mean ± SEM, ns, not significant.

To confirm *dbr* implication in memory, we analyzed the effect on memory of *dbr* silencing mediated by RNA interference (RNAi) [27], [28]. Because RNAi-mediated knockdown might be prone to off-target effects, in addition to the *dbr-RNAi-A* construct obtained from the Vienna Drosophila RNAi Center (Austria), we designed another RNAi construct (see Materials and Methods) and analyzed 2 distinct transgene insertions (*RNAi-B1* and *RNAi-B5*). Before performing behavioral experiments, we assessed *dbr* mRNA targeting by the distinct specific RNAi constructs. For that purpose, we used the elav-Gal4 driver (elav) that drives pan-neuronal expression of UAS*^GAL4^*-regulated transgenes [27]. *dbr* expression in fly heads was quantified by real-time PCR. The data showed a 45%, 50% and 55% *dbr* mRNA decrease in *elav/+;RNAi-A/+*, *elav/+;RNAi-B1/+* and *elav/+;RNAi-B5/+* flies, respectively, compared to levels observed in *elav/+* control flies (Figure 3A, left panel). In contrast, *+/RNAi* flies displayed *dbr* mRNA levels similar to wild type (Figure 3A, right panel). Altogether, the results indicate that either RNAi construct targets efficiently *dbr* RNA, leading to a decrease in *dbr* mRNA expression.

Although immunohistochemistry analyses of *72Y* fly brains did not reveal any gross structural defect in the anatomy of the MBs (data not shown), we cannot exclude that the LTM impairment observed in these flies is not caused by more subtle developmental defects. In order to avoid potential developmental defects that could affect memory performance, we investigated the effect of *dbr* RNAi expression restricted to adulthood by taking advantage of the conditional Gal4-Switch/UAS*^GAL4^* system [29] under the control of MB247 sequences (MB-Sw) [30]. The MB247 enhancer drives a specific expression in a subset of α/β and γ neurons [31]. We first verified that RNAi-expressing flies exhibited normal response to electric shocks (Figure 3B) and olfactory sensitivity (Figure 3C and D). When memory tests were performed immediately after a single conditioning session, flies expressing the *dbr*-*RNAi-A* construct in the adult MBs displayed normal scores (Figure 3E). When memory was tested 2 hrs after a single conditioning cycle, flies expressing *dbr* RNAi did not display memory scores significantly different from the controls (Figure 3F). Taken together, the data show that neither learning nor STM are sensitive to *dbr* RNAi expression. To further analyze ARM, consolidated memory was assessed after massed conditioning. Memory scores at 24 hrs were not significantly decreased when *dbr* RNAi was expressed (Figure 3G), showing that ARM is not affected. LTM analysis after five spaced conditioning cycles revealed that flies expressing *dbr* RNAi displayed significantly lower scores than their respective controls (Figure 3H). Importantly, when flies were not fed with RU, *MB-Sw/RNAi* flies exhibited normal performance at 24 hrs after spaced conditioning (Figure 3I, hatched bars), whereas, as previously observed, when fed with RU, *MB-Sw/RNAi* flies exhibited an impaired LTM (Figure 3I, gray bars). These results show that the observed LTM decrease is RU-specific and is thus caused by the Gal4-Switch dependent induction of *dbr* RNAi expression. In conclusion, the data establish that transient expression in the adult MBs of RNAi directed against *dbr* impairs LTM, while neither STM nor ARM are affected. Therefore *dbr* is physiologically required for LTM processing.

### Behavioral analysis of *dbr* overexpression in the adult MBs

**Figure 4:**
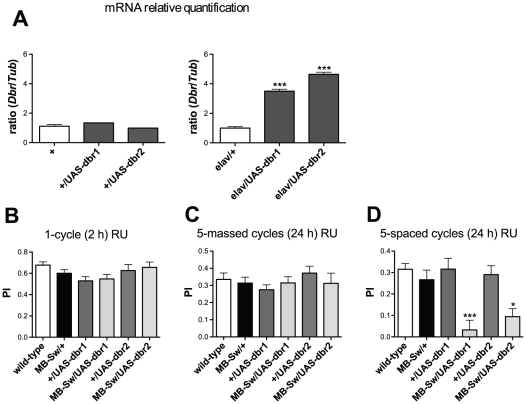
*dbr* overexpression in the adult MBs leads to a LTM-specific defect. (A) *dbr* mRNA expression analyses. Total RNA was extracted from fly heads, submitted to DNase treatment, and further reverse transcribed with oligo(dT) primers. Resulting cDNA was quantified by PCR using tubulin (*Tub*) expression as a reference. Results are shown as ratios relative to the values observed for wild-type (+, left panel, *t* test, *p*>0. 5, n = 2) and *elav/+* (right panel, *t* test, *p*<0.0001, n≥2) control flies. (B–D) Behavioral analysis. To achieve *dbr* overexpression in the adult MBs, flies were fed with RU for 48 hrs prior to conditioning and in (C and D), also for 24 hrs before testing. (B) STM analysis 2 hrs after a single conditioning cycle (ANOVA, *p* = 0.3049, *n*≥11). (C) ARM analysis 24 hrs after five massed conditioning cycles (ANOVA, *p* = 0.6229, n≥14). (D) LTM analysis 24 hrs after five spaced conditioning cycles (ANOVA, *p*<0.0001, *n*≥15). Bars indicate Mean ± SEM.

To further analyze *dbr* involvement in LTM, we decided to overexpress *dbr* in the adult MBs. We first verified that the *UAS-dbr* construct that we generated (see Materials and Methods) could efficiently lead to *dbr* overexpression. We analyzed 2 distinct insertions of the *UAS-dbr* transgene (*UAS-dbr1* and *UAS-dbr2*). Quantitative PCR analyses revealed that in the absence of a Gal4 driver (*+*/*UAS-dbr*), *dbr* mRNA levels were similar to the wild-type control (Figure 4A, left panel). In contrast, *dbr* mRNA was increased 3.5 to to 4.6-fold in *elav/+;UAS-dbr/+* fly heads compared to levels observed in *elav/+* control flies (Figure 4A, right panel).

Behavioral experiments were conducted with flies expressing either *UAS-dbr* transgene in the adult MBs under the control of the MB-Sw driver. The data showed that flies overexpressing *dbr* in the adult MBs exhibited a strong LTM defect, whereas ARM and STM were normal (Figure 4B, C and D). Taken together, the results show that both *dbr* silencing and *dbr* overexpression in the adult MBs lead to an LTM-specific impairment.

## Discussion

We report here the identification of *dbr* as a new LTM mutant. We show that enhancer-trap P(Gal4) inserted nearby the *dbr* gene lead to Gal4-dependent expression in the MBs, a major center of olfactory memory. The MB247 driver used to affect *dbr* levels in our study leads to a specific expression in the MB α/β and γ neurons [31], consistent with additional reports showing that these neurons are involved in aversive olfactory LTM [32], [33], [34].

Several reports have shown that *dbr* is involved in various developmental processes [35], [36], [37], [38]. Importantly, the use of conditional silencing in our study reveals that the LTM-specific impairment observed is not caused by a developmental defect, demonstrating that *dbr* is physiologically involved in LTM processing.

Dbr does not exhibit any obvious homology with known proteins, and its molecular function is unknown. Dbr has been shown to interact with the F-box protein Slimb, an ubiquitin ligase [26]. In cooperation with Slimb, Dbr induces the polyubiquitination of phosphorylated Ci-155, a transcription factor that mediates Hedgehog signaling [26]. Interestingly, similar to Dbr, Slimb has been implicated in LTM formation [16], thus pointing to a role for ubiquitination in LTM processing. These observations are reminiscent of a previous study showing that the highly conserved ubiquitin ligase Neuralized (Neur) is involved in LTM [39]. Neur is expressed in the adult MB α/β neurons and is a limiting factor for LTM formation: loss of one copy of *neur* gene results in significant LTM impairment whereas Neur overexpression results in a dose-dependent enhancement of LTM [39]. In contrast, both *dbr* silencing and *dbr* overexpression in the adult MBs generate a LTM defect, showing that *dbr* levels must be precisely regulated to support normal LTM, a situation similar to previous reports describing LTM-specific mutants [17], [18].

Interestingly, *dbr* is specifically required for LTM since it is dispensable for earlier memory phases. LTM is the only form of memory that relies on *de novo* protein synthesis, a process thought to underlie synaptic plasticity. Since proteins are the molecular actors that mediate signal transduction, protein synthesis as well as protein degradation must be important for plasticity and memory. Indeed, regulated proteolysis plays a critical role in the remodeling of synapses [40]. Regulated proteolysis is achieved by two major systems in eukaryotic cells: the proteasome and the lysosome [41]. The lysosome degrades most membrane and endocytosed proteins. Owing to their large surface-to-volume ratio, the degradation of membrane proteins such as receptors by the endocytic/lysosomal pathway must be especially efficient and tightly regulated in neurons [41]. Whereas several studies have implicated the proteasome in LTM in *Aplysia* [42], [43], [44], in the crab [45] and in mammals [46], [47], [48], less is known about the implication of the lysosome in this process. It has been suggested that Neur is implicated in both the proteasome and the lysosome degradation pathways [49]. Dbr is involved in protein degradation [26], [50], and has been characterized as a component of the multivesicular bodies (MVB), an actor of the lysosome pathway [26]. Ubiquitinated receptors undergo endocytosis and become incorporated into endosomes that are in turn sequestered into MVB. Subsequently, the MVB membrane becomes continuous with lysosomes leading to degradation of the receptor [51]. Although we cannot rule out that *dbr* could be implicated in LTM via another pathway, we suggest that its function in LTM takes place through the lysosomal protein degradation pathway.

## Materials and Methods

### Fly strains and culture

*Drosophila melanogaster* wild-type strain Canton-Special (CS) and mutant flies were raised on standard medium at 18°C, 60% humidity in a 12:12 hrs light:dark cycle. All strains used for memory experiments were outcrossed to the CS background. To induce the expression of UAS-RNAi and UAS-cDNA constructs, the gene-Switch system was used as previously described [30]. Flies aged between 1 and 2 d were kept on RU486-containing medium (RU) (Mifepristone, SPI BIO) for 2 d prior to conditioning, and also for 24 hrs after when memory was tested at 24 hrs. An appropriate amount of a RU stock solution (10 mM in 80% Ethanol) was mixed into molten food at 65°C to a final concentration of 200 µM.

### Behavior analyses

Flies were trained with classical olfactory aversive conditioning protocols as described in [3]. Training and testing were performed at 25°C with 80% humidity. Conditioning was performed on samples of 25–35 flies aged between 3 and 4 d with 3-octanol (>95% purity; Fluka 74878, Sigma-Aldrich) and 4-methylcyclohexanol (99% purity; Fluka 66360, Sigma-Aldrich) at 0.360 mM and 0.325 mM, respectively. Odors were diluted in paraffin oil (VWR international, Sigma-Aldrich). Memory tests were performed with a T-maze apparatus [2]. Flies could choose for 1 min between 2 arms each delivering a distinct odor. An index was calculated as the difference between the numbers of flies in each arm divided by the sum of flies in both arms. A performance index (PI) results from the average of two reciprocal experiments.

### Shock sensitivity and olfaction tests

For shock sensitivity test, two barrels, identical to that normally used for conditioning, were connected to each other by a Plexiglas dish. Flies were trapped in the middle, and were allowed for 1 min to move towards either barrel, one of which was electrified as for conditioning. For odor avoidance tests, flies were treated in the barrel as for associative conditioning, except that presentation of the second odor was omitted [52]. Treated flies were transported to the choice point of the T-maze immediately after training and allowed to choose between the second odor and air. PI were calculated as for memory tests.

### Statistical analyses

Scores were analyzed using two-tailed unpaired Student's *t* tests to compare two groups (Figures 1, 2, 3A and I, 4A). To compare more groups (Figures 3B-H and 4B–D), scores resulting from all genotypes, excluding the wild-type CS, were analyzed using one-way ANOVA followed by the Newman–Keuls multiple comparisons test if significant at *p*<0.05.

### PCR-Rescue

Genomic DNA adjacent to the P-element insertion was isolated by inverse PCR as described in http://www.fruitfly.org/about/methods/inverse.pcr.html. Digestions were performed in parallel with Sau3A I, HinP1 I and Msp I. All constructs were verified by sequencing (Eurofins).

### UAS-Gal4 constructs

The *dbr-RNAi-A* line was obtained from the Vienna Drosophila RNAi Center (Austria)(construct ID 7281). To construct *dbr-RNAi-B*, a 559 bp fragment was amplified from CS genomic DNA with the following oligonucleotides (capital letters correspond to *dbr* sequences): 5′-cgctagtctagaCCACGTGCCGGAGTCCGGAAA-3′ and

5′-cgctagtctagaCCTGTCCGGTACGCATGGCTTT-3′. The resulting fragment was cloned into pGEMT, and subsequently cloned into pWIZ [53]. This latter construct was injected into *w^1118^* embryos (BestGene Inc.). Two distinct transformants *dbr-RNAi-B1* and *dbr-RNAi-B*5 were used for behavioral analyses. To construct the *UAS-dbr* lines, a full-length *dbr* cDNA from BGDP (clone LD26519) was digested with Xho I and Bcl I and further inserted into the pCaSpeR-UAS vector digested with Xho I and Bgl II. Two distinct transformants, *UAS-dbr1* and *UAS-dbr2*, were used for behavioral analyses. All constructs were verified by sequencing (Eurofins).

### Immunohistochemistry

Freshly dissected brains of adult flies were processed for immunochemistry as described previously [54]. Primary antibodies were mouse anti-FasII at 1∶400 (1D4; Developmental Studies Hybridoma Bank, University of Iowa, Iowa City, IA) and rabbit monoclonal anti-GFP at 1∶200 (G10362, Invitrogen). Secondary antibodies were Alexa Fluor 488-conjugated anti-rabbit at 1∶400 (Invitrogen) and Alexa fluor 594-conjugated anti-mouse at 1∶400 (Invitrogen).

### Quantitative PCR analysis

Flies expressing *dbr* RNAi and *dbr* cDNA, respectively, under the control of the elav-Gal4 driver were raised at 25°C until aged 1 to 3 d. Total RNA was extracted from 30 female fly heads with RNeasy Plant Mini Kit (Qiagen) and further submitted to DNase I treatment (Biolabs). Reverse transcriptase reactions were carried out with the SuperScript III First-Stand Kit (Invitrogen) according to the manufacturer instructions. 1.5 µg of total RNA was reverse transcribed with Oligo(dT)_20_ primer. We compared the level of *dbr* cDNA to that of the *α-Tub84B* cDNA (CG1913) used as a reference. Specific primers were designed based on sequence data from the Genebank database. Amplification was performed using a LightCycler 480 (Roche) in conjunction with the SYBR Green I Master (Roche). Reactions were carried out in triplicate for at least 2 dilutions of each cDNA, and two to four independent experiments were performed. 45 cycles were conducted using 0.5 pmol of each primer under a 2-step PCR with an annealing-elongation temperature of 60°C. Specificity of amplification products was assessed by melting curve analysis and the control of each product size by running a sample of the product on agarose gel. Expression relative to *tub* is expressed as a ratio (2^-ΔΔCp^). A ratio of 1 represents the relative expression observed in control flies.

Oligonucleotides used in this study were:

Tub-F 5′-TTGTCGCGTGTGAAACACTTC-3′

Tub-R 5′-CTGGACACCAGCCTGACCAAC-3′

Debra-F 5′- AAGAAAAGGGAGGATGAAGAAC -3′

Debra-R 5′- ACATGCGAATCAACCGATATAG -3′

PCR products were 81 bp to 125 bp in length, respectively.
